# Do we need a regulatory path for HIV post‐exposure prophylaxis?

**DOI:** 10.1002/jia2.26449

**Published:** 2025-06-26

**Authors:** Veronica Miller, Robin Schaefer

**Affiliations:** ^1^ Forum for Collaborative Research UC Berkeley School of Public Health Washington DC USA

1

HIV post‐exposure prophylaxis (PEP) is an important but underutilized HIV prevention tool. The scientific rationale for PEP is based on (1) the known mechanism of action of antiretrovirals in interfering with HIV replication and establishment of infection, (2) animal and pharmacokinetic/pharmacodynamic studies, and (3) studies among healthcare workers and other populations treated with zidovudine‐based PEP, resulting in initial PEP guidelines [1]. Since these early studies, no comparative PEP efficacy trials have been conducted. Despite the absence of efficacy data, PEP guidelines by the US Centers for Disease Control and Prevention (CDC), World Health Organization (WHO) and other agencies have been updated based on the availability of more potent and tolerable regimens, further supportive animal studies, extrapolation from treatment studies, and non‐randomized research [1, 2]. Contemporary PEP recommendations consist of a 28‐day, three‐drug oral regimen. However, other than zidovudine for preventing vertical transmission, no antiretroviral product has a labelled indication for PEP. It is thus implemented “off‐label” based on recommendations by normative bodies.

Adherence to the recommended 28‐day oral PEP regimen is often suboptimal [3, 4] and incomplete adherence may contribute to HIV seroconversion [5]. New long‐acting antiretroviral drug formulation could thus improve PEP effectiveness and impact. However, demonstrating the efficacy (or effectiveness) of new products as PEP faces considerable challenges, including the low likelihood of HIV acquisition following PEP initiation and an effective standard‐of‐care PEP regimen (as discussed by Ortblad et al. in this supplement [6]). Given the consensus about existing PEP efficacy, placebo‐controlled trials are not ethical, and active‐control randomized non‐inferiority trials may require unfeasibly large sample sizes. Considering these challenges, do we need a regulatory path for PEP, and if so, what would it look like?

An approved indication from a trusted regulatory authority implies rigorous science, review and benefit versus risk considerations for that indication, transparently debated in public—or with public access to the process. It builds confidence among policymakers, healthcare providers and users. An approved indication authorizes the marketing of that product for that indication, possibly resulting in improved awareness and access. A labelled indication may facilitate coverage through health insurance or public healthcare systems, further improving access. More available products with a PEP indication would increase product choice, aligning with user preferences and needs and potentially improving uptake and effective use. Finally, regulatory approvals for a PEP indication may improve global access through regulation by reliance. In this process, a regulatory authority utilizes the assessment of another trusted authority when evaluating a product. This is of particular benefit to regulatory authorities with more limited resources and can accelerate approval timelines and increase the availability of products.

Various drugs are used as PEP to reduce infectious disease risks. Oseltamivir phosphate was approved for influenza PEP based on randomized household transmission studies [7]. By the late 2000s, it was widely approved—including in the United States [8], European Union [9] and South Africa [10]—and available in over 80 countries [11]. Approval by the European Medicines Agency facilitated WHO prequalification in 2009 [12]. In contrast, doxycycline is being integrated into clinical practice in the United States as PEP for non‐HIV sexually transmitted infections (STIs) (doxy‐PEP) without regulatory PEP indication; rather, it is recommended by the US CDC for some individuals based on randomized clinical trials [13]. In other countries, such as the UK [14], doxy‐PEP is not recommended, and it remains to be seen how uptake by providers and patients will evolve over time and across geographies. However, these examples may not be generalizable to HIV PEP as the transmission rate and incidence of influenza and non‐HIV STIs are higher than those of HIV, and these clinical studies were able to compare the PEP agent against a placebo or no PEP.

Contraception may illustrate possible regulatory pathways for HIV PEP. US Food and Drug Administration (FDA) guidance for hormonal contraception recognizes that (1) placebo‐controlled trials are not feasible, (2) expected pregnancy rates are high in the absence of contraception, and (3) the treatment effect is high [15]. Together with the understanding of drug mechanisms of action, this justifies single‐arm, open‐label trials, with comparison to historical controls to establish efficacy measured by the Pearl Index (the number of unintended pregnancies per 100 years of exposure) and life table analyses [15]. This ensures efficient drug development for increased product choice. Emergency contraception could be considered analogous to HIV PEP. For example, ulipristal acetate was approved by the US FDA for emergency contraception in 2010 based on an open‐label single‐arm and a single‐blind comparative clinical trial [16]. In both trials, primary analyses compared the observed pregnancy rate among those who received emergency contraception with the expected pregnancy rate.

Assuming ample pharmacokinetic, pharmacodynamic and safety evidence, the major hurdle for an HIV PEP labelled indication is demonstrating efficacy: “Does the drug prevent HIV after exposure?” Applying the emergency contraception regulatory pathway, a study would not address “Is the new drug better than existing ones?” but demonstrate no or hardly any HIV acquisitions in settings with at least a modest number of expected acquisitions in the absence of PEP. A sub‐analysis might be considered for exposures with known‐serostatus index cases (e.g. in healthcare settings). It might be useful for studies to offer a standard‐of‐care 28‐day oral regimen option. This would not be intended to generate comparative effectiveness evidence but data on preferences and acceptability. Further secondary outcomes might include adherence, return to follow‐up visits, patient satisfaction, and adverse events.

There are clear potential advantages in a regulatory PEP indication, improving trust by providers and users, increasing access, and resulting in more choices for those who could benefit from PEP. This, in turn, can improve the uptake and effective use of PEP. Whether the pathways for a PEP indication proposed here or elsewhere in this supplement are acceptable to communities and regulatory authorities requires input from all stakeholders. Such multistakeholder processes to facilitate consensus have supported novel clinical trial designs for HIV pre‐exposure prophylaxis [17] and they should be used to clarify critical issues around PEP, involving regulators, communities, ethicists, researchers, and industry. Through such collaboration, the untapped potential of PEP can be realized.

## COMPETING INTERESTS

The Forum for Collaborative Research receives unrestricted grants from the pharmaceutical industry, including from companies involved in the development of antiretroviral drugs (ViiV Healthcare, Merck and Gilead Sciences). These grants are provided to the organization and are not specifically linked to the present work. The authors (as individuals) have no relevant financial or non‐financial interests to disclose.

## AUTHORS’ CONTRIBUTIONS

VM produced the first draft of the manuscript. VM and RS finalized the manuscript.

## FUNDING

This work was supported, in whole or in part, by the Gates Foundation (INV‐045445). Under the grant conditions of the Foundation, a Creative Commons Attribution 4.0 Generic License has already been assigned to the Author Accepted Manuscript version that might arise from this submission.
